# Progression risk assessments of individual non-invasive gastric neoplasms by genomic copy-number profile and mucin phenotype

**DOI:** 10.1186/s12920-015-0080-6

**Published:** 2015-02-18

**Authors:** Diem Thi-Ngoc Vo, Takahisa Nakayama, Hiroto Yamamoto, Ken-ichi Mukaisho, Takanori Hattori, Hiroyuki Sugihara

**Affiliations:** Department of Pathology, Division of Molecular and Diagnostic Pathology, Shiga University of Medical Science, Otsu, 520-2192 Japan

**Keywords:** Copy-number variation, Microarray, Non-invasive neoplasia, Gastric cancer, Mucin phenotype, Array-based comparative genomic hybridization, Unsupervised hierarchical clustering

## Abstract

**Background:**

Early detection and treatment of non-invasive neoplasms can effectively reduce the incidence of advanced gastric carcinoma (GC), but only when the lineage is continuous between non-invasive and advanced tumours. Although a fraction of non-invasive neoplasms progress to invasive GC, it is difficult to identify individual progression-prone non-invasive neoplasms. To classify non-invasive gland-forming gastric neoplasms into clusters of different levels of progression risk, we applied mucin phenotyping and genomic DNA microarray analyses to intramucosal gland-forming gastric neoplasms.

**Methods:**

Formalin-fixed, paraffin-embedded tissues from 19 non-invasive and 24 invasive gland-forming neoplasms were obtained via endoscopic submucosal dissection or surgical excision. According to the Vienna classification, intramucosal neoplasms were classified as low-grade or high-grade non-invasive neoplasms (LGNs [category 3] and HGNs [category 4], respectively) or invasive carcinomas (intramucosal GCs and mucosal parts of submucosal or deeper GCs [category 5]). Neoplastic lesions were characterized by mucin phenotypes determined using monoclonal antibodies against MUC2, MUC5AC, MUC6, and CD10. Genomic DNA samples from mucosal neoplasms were subjected to array-based comparative genomic hybridization and subsequent unsupervised, hierarchical clustering with selected large-sized genes.

**Results:**

There was no significant difference in mucin phenotype between HGNs/LGNs and invasive carcinomas. The clustering classified samples into stable, unstable, and intermediate. The histological tumour grade or mucin phenotype of non-invasive neoplasms did not correlate with the clustering results. Each cluster may represent an independent lineage of different outcome because the size distribution of non-invasive tumours among the 3 clusters almost overlapped. In contrast, the unstable cluster alone included invasive carcinomas.

**Conclusions:**

These findings suggest that the outcome of individual tumours is not stochastically determined but can be predicted from the genomic copy-number profile even at the non-invasive stage. Non-invasive neoplasms of the unstable clusters, which accounted for 21% of non-invasive neoplasms, may progress to invasive carcinomas, whereas those of stable cluster may not.

**Electronic supplementary material:**

The online version of this article (doi:10.1186/s12920-015-0080-6) contains supplementary material, which is available to authorized users.

## Background

Gastric carcinoma (GC) is one of the most common malignant tumours and has the fourth highest mortality rate worldwide [1]. Endoscopic examination for early GC detection has been refined by recent technical developments such as narrow band imaging and magnifying observation endoscopes [2-5]. Therefore, endoscopically resected, non-invasive gland-forming gastric neoplasms are more frequently encountered during pathological examinations. Increased detection and treatment of non-invasive neoplasms may contribute to a reduction of GC-associated mortality, but only if the genetic lineage is continuous from the non-invasive neoplasms to advanced GCs.

Regarding the outcome of gastric non-invasive neoplasms, Western and East Asian follow-up studies conducted between 1987 and 2008 reported that low-grade neoplasms (LGNs) and high-grade neoplasms (HGNs) progressed to invasive carcinomas at frequencies of 0–23% and 10–85%, respectively [6-15]. These varying incidences may reflect varied pathological criteria for differentiating intramucosal neoplasms and different mean follow-up times. After standardizing the criteria according to an international consensus (Vienna classification), recent follow-up studies indicated that approximately 10% of LGNs progressed to invasive carcinomas [12,15].

However, it is difficult to specify individual progression-prone non-invasive neoplasms without a progression-specific marker. When the morphological grade was used as such a marker, long-term follow-up studies demonstrated that almost a third of HGNs remained unchanged despite their high-grade histology [12]. Therefore, histological grade has limited value when assessing the progression risk of individual tumours. In the present study, we used 2 more specific lineage markers: mucin phenotype, as a cell type-specific gene expression marker, and genomic DNA copy-number profile, as a genetic lineage marker.

The mucin phenotype, determined by the expression pattern of mucin core proteins (encoded by the MUC gene family) and another protein, CD10, expressed in the brush border of small intestinal enterocytes, is used to classify cell types as gastric and intestinal [16,17]. In normal tissues, different phenotypes are determined epigenetically, and stably inherited from cell to cell. This heritability of phenotypes can be used in lineage-specific progression risk assessment studies. Previous studies reported that non-invasive neoplasms commonly progressed to invasive adenocarcinoma in the gastric lineages, but rarely in the intestinal lineages [14,16-18]. However, during tumour progression from an early to an advanced stage, metaplastic intestinal-type expression [19,20] and/or the progression-related loss of gastric-type expression [17,20] can occur.

As a genetic lineage marker, we focused on overall genomic DNA copy-number profiles, which are determined by array-based comparative genomic hybridization (aCGH). These profiles are unique for individual neoplasms because they include random alterations of genes that are non-essential for carcinogenesis and accumulate over time based on genetic instability. The samples were classified based on overall similarities in the DNA copy-number profiles by an unsupervised hierarchical cluster analyses.

In the present study, we compared two lineage analyses using identical non-invasive and invasive gastric tumours to investigate whether they enabled the prediction of the progression risks of individual non-invasive gastric neoplasms. The results indicated that the genetic, but not the phenotypic markers could predict the progression risk of individual non-invasive gastric neoplasms.

## Methods

### Tissues samples

The Institutional Review Board on Medical Ethics at Shiga University of Medical Science approved the study on the condition that the materials used remained anonymous (Permission number: 14-57-5 on 16 November 2013). Written informed consent was not required for this retrospective study that detected acquired genomic changes in archival materials alone.

**Figure 1:**
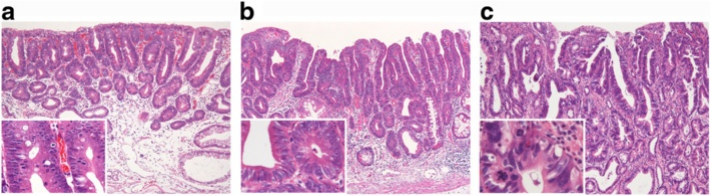
**Representative haematoxylin and eosin (H & E) images of neoplasms in groups A, B, and C. (a)** Group A (Vienna category 3: non-invasive low-grade neoplasia with dark, slender nuclei); **(b)** group B (Vienna category 4: non-invasive high-grade neoplasia with ovoid vesicular nuclei and prominent nucleoli (carcinoma in situ)); **(c)** group C (Vienna category 5: invasive neoplasia with pleomorphic, hyperchromatic nuclei and structural atypia). The inset in each image shows a high power view.

Formalin-fixed, paraffin-embedded (FFPE) tissues from 19 non-invasive and 24 invasive intramucosal gastric neoplasms, which were endoscopically or surgically resected from 38 patients, were obtained between 2009 and 2013. Of the invasive carcinomas, tumours comprising a ≥50% tubular-forming component, which included at least a portion of well-differentiated tubular area, were used in this study. Intramucosal lesions were classified histologically into 3 groups according to the Vienna classification [21]: group A (non-invasive low grade neoplasia [category 3]), group B (non-invasive high grade neoplasia [category 4]), and group C (invasive tubular adenocarcinoma [category 5]), as shown in Figure 1. Group C was subdivided into Cm (intramucosal invasive carcinoma) and Cd (intramucosal part of submucosal or deeper invasive carcinoma). Pathological stages were determined according to the Japanese classification of GC [22].

### Immunohistochemistry

Mucin phenotypes were evaluated immunohistochemically using monoclonal antibodies (Novocastra, Newcastle, UK) against the goblet cell mucin MUC2 (Ccp58; 1:100 dilution), gastric-foveolar mucin MUC5AC (CLH2; 1:100), pyloric-gland mucin MUC6 (CLH5; 1:100), and brush border CD10 (56C6; 1:100). Immunohistochemical staining was performed using an automated Ventana Discovery XT system (Tucson, AZ, USA) with heat pre-treatment and a universal DAB detection kit (Ventana). The used sections included areas corresponding to those obtained by laser microdissection for DNA extraction (described in the Genomic DNA extraction methods).

**Figure 2:**
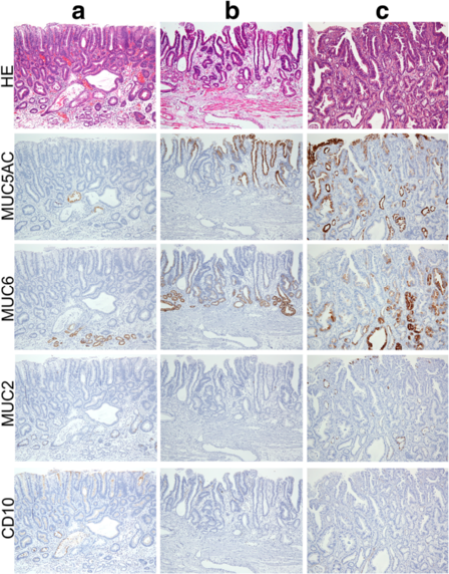
**Representative H&E images and different marker expressions. (a)** Low-grade non-invasive neoplasia (group A) complete intestinal type (I-type). Tumour glands are rarely and frequently positive for MUC2 and CD10, respectively, and negative for MUC5AC and MUC6. In contrast, residual normal glands are positive; **(b)** High-grade non-invasive neoplasia (group B), complete gastric type (G-type). Tumour glands are positive for MUC5AC and MUC6, and negative for MUC2 and CD10; **(c)** invasive intramucosal tubular adenocarcinoma (group C), gastrointestinal type (GI-type). Tumour glands are positive for MUC5AC, MUC6, and MUC2, and negative for CD10.

The extent of MUC2, MUC5AC, MUC6 and CD10 expression was scored according to the percentage of stained neoplastic cells as follows: (−), 0% to <5% positive cells; (+), some positive cells, (≥5% to <30%); (++), well-defined areas of positive cells (≥30% to <60%) and (+++), extensive areas of positive cells (≥60%). According to the gastric markers (MUC5AC and/or MUC6) and intestinal markers (MUC2 and/or CD10) scores, each neoplasm was phenotypically classified as pure gastric type (G-type), mixed type (GI-type), complete intestinal type (I-type) or null type (N-type) as described previously [17] (Figure 2).

### Genomic DNA extraction

Tumour and normal gland (reference) samples were obtained from 5-micron-thick tissue sections using a laser microdissection system (LMD6000; Leica Microsystems, Wetzlar, Germany). Each sample was dissected from an area of ≥6 mm^2^. In tumour samples, neoplastic cells comprised 90% of the total cell count. These cells were then digested in a 200-mg/mL proteinase K (P2308, Sigma-Aldrich, St. Louis, USA) solution for 70 ± 2 h at 37°C prior to the phenol/chloroform DNA extraction. Corresponding tumour areas were assessed for the immunohistochemical staining of mucin and other markers as described above. DNA quality was assessed based on the A260/A280 ratio (cut-off. >1.5) and A260/A230 ratio (cut-off. > 1.0) and by the presence or absence of double-stranded DNA.

### Whole genome amplification (WGA)

Sample DNA was amplified using the GenomePlex Whole Genome Amplification Kit (WGA2 Kit; Sigma, St. Louis, MO, USA) according to the manufacturer’s protocol [23].

### Array CGH

For genomic DNA analysis, a 60-mer oligonucleotide aCGH (Agilent, Santa Clara, CA, USA) was used, according to the manufacturer’s instructions [24]. The Genomic DNA Enzymatic Labelling Kit (version 7.2, 2012) was used for small-size LGNs, whereas the Genomic DNA ULS Labelling Kit (non-enzymatic labelling) was used for massive and invasive cancer samples (protocol CGH_107_Sep09 and Grid: 021924_D_F_20100501 and protocol CGH_107_Sep09 and Grid: 021924_D_F_20111015). Briefly, tumour and control DNAs were labelled with Cy5 and Cy3, respectively, followed by competitive hybridization to microarrays (SurePrint G3 CGH Microarray 8 × 60 K, GPL10152 62,976 probes). The tumour-to-reference fluorescence intensity ratio (T/R) was calculated from the hybridized array images obtained using a DNA microarray scanner (Feature Extraction software 10.7.3.1). Agilent CGH Analytics Software was used to visualize, detect, and analyse chromosomal patterns within the microarray profiles. The UCSC Genome Browser was applied according to the latest resource content: hg19 assembly - Design ID 021429 (GRCh Build 37). Copy-number alterations (CNAs) were defined as gains and losses when base 2 logarithm of the T/R ratios were >0.3219 and <−0.3219, respectively. The microarray data were registered in the Gene Expression Omnibus (GEO) database (Accession number: GSE60116).

### Clustering algorithm

To enhance the signal-to-noise ratios in hybridization analyses, we averaged the T/R ratio of the probes within each gene prior to performing cluster analyses. To classify samples based solely on genome-wide resemblances in the gene copy-number gain/loss patterns, unsupervised hierarchical cluster analyses were performed using a free software programme (Cluster 3.0, version 1.52 and TreeView, version 1.1.6r2) [25,26]. We selected genes by size; for larger genes, more probes were included within the genes, and the noise cancelling effect was expected to increase by averaging the probe data. We attempted repeated clustering using genes ranging from 372 genes containing ≥10 probes to 9,615 genes containing ≥2 probes. The optimal gene size was determined as the largest gene that fulfilled the following standards: first, ≥2 identical tumour samples were located at neighbouring positions in the tree diagram of cluster analyses because these samples have more common CNAs during carcinogenesis than any other tumours [27]; second, each cluster’s sample constitution becomes constant. The clustering condition was set to a complete linkage (maximum of distance metric on similarities) and the uncentred correlation distance (distance measures based on modified Pearson’s correlation).

### Genes exhibiting significantly different CNAs between clusters

We applied *Welch’s t*-test with Bonferroni correction between the averaged log_2_ (T/R) values (reflecting averaged copy-numbers) of total aggressive and total stable tumour samples for 14,753 protein-encoding genes (Office Excel 2013; Microsoft, Redmond, WA, USA).

### Microarray data validation by quantitative polymerase chain reaction (PCR)

Remaining DNA samples (tumour and reference) from 8 randomly selected sample pairs, used in aCGH analyses, were subjected to quantitative PCR. Primers (Additional file 1: Table S1) were designed using Primer3 software (http://bioinfo.ut.ee/primer3-0.4.0/). As an internal standard, we used one set of primers that was specific for chr15:51481794–51481853 and selected from the genomic DNA portion with few gains or losses. PCR was carried out in a final volume of 10 μL using the LightCycler Nano following the manufacturer’s instructions (Roche, Basel, Switzerland). Briefly, the reaction mixture consisted of 500 nM of each forward and reverse primer, 10 ng of DNA sample and 1 × FastStart Essential DNA Green Master mix (Roche). PCR was performed in duplicate using the following conditions: denaturing at 95°C for 10 min, followed by 45 cycles of PCR at 95°C for 10 s, annealing and elongating at 60°C for 30 s. Cq value was determined by selecting the second derivative maximum method equipped on the LightCycler Nano.

### Statistical analysis

The differences in CNA for each gene and in phenotypic expression among groups A, B, and C were statistically assessed in an unequal sample-size *t*-test (*Welch’s t*-test) [28]. A bilateral *p*-value of ≤0.05 was considered statistically significant. For multiple comparisons, the *t* test was adjusted subsequently using the Bonferroni correction [23,28] (Microsoft Office Excel 2013). To assess trend differences in either mucin phenotypes or CNA accumulations between the 2 groups, a Fisher’s exact test (2 × 2 contingency tables) or the Cochran–Armitage test (for 2 × κ contingency tables) was performed (Excel Statistics for Windows, 2012 Edition, Social Survey Research Information Co., Ltd., Tokyo, Japan).

## Results

### Histology and mucin phenotypic expressions among groups A, B, and C

Of the 43 tumours, 7 were categorized as group A, 12 as group B, 8 as group Cm, and 16 as group Cd. The Cd group tumours comprised 3 submucosal and 13 advanced cancers. The average ages of patients did not differ between groups A and B or groups Cm and Cd (*p* = 0.48, *p* = 0.50, respectively), but they were higher in group C than in group A/B (*p* = 0.0002). Other clinicopathological features are summarized in Table 1 and Additional file 1: Tables S2a and S2b. Multiple non-invasive neoplasms that incidentally coexisted with main lesions were also analysed and marked as pairs 1–5.

**Table 1:** **Clinicopathological features of tumour samples in groups A, B, and C according to the Vienna classification**

**Group**	**Group A**	**Group B**	**Group C**	
			**Cm**	**Cd**
Sex (M:F)	4:3	7:5	6:2	12:4
Age (years)				
Range	59 – 81	41 - 77	59 – 92	64 – 88
Mean ± SD	66.5 ± 10.0	62.1 ± 9.7	74.25 ± 10.4	77.1 ± 7.4
Size (mm)				
Range	2 – 12	3 – 30	15 – 40	15 – 100
Mean ± SD	5.7 ± 3.1	9.4 ± 7.9	27.1 ± 7.8	51.1 ± 27.1

Cm, intramucosal invasive carcinoma; Cd, intramucosal part of submucosal or deeper invasive carcinoma.

Concurrent intramucosal lesions sampled from 5 patients were comprised 5 tumour pairs: A3–A7, B5–B12, A4–B11, Cd1–Cd8 and A2–Cd12. The distance between the lesions in each pair ranged from 1.5–7 cm.

The tumour sizes of the intra-mucosal lesions in groups A, B, Cm and Cd were 5.7 mm (range, 2–12 mm), 9.4 mm (range, 3–30 mm), 27.1 mm (range, 15–40 mm) and 51.1 mm (range, 15–100 mm), respectively.

**Figure 3:**
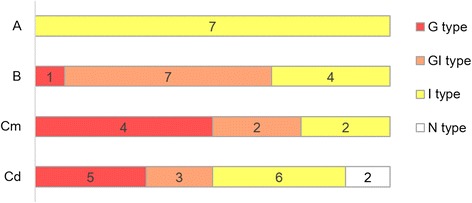
**Phenotypic constitution of tumours in groups A, B, and C.** The mucin phenotype is classified into gastric (G) type, gastrointestinal (GI) type, intestinal (I) type and null (N) type. The numerals in the colour bars indicate numbers of samples in each group. Regarding Cm and Cd, see the legend for Table 1.

The mucin phenotype analyses are shown in Figure 3 and Additional file 1: Table S3. None of the 7 tumours in group A expressed gastric phenotypic markers (completely intestinal phenotype), whereas 8 of 12 tumours in group B did. Among the 24 tumours of group C, 14 expressed gastric markers, 8 expressed intestinal markers and 2 had no mucin expression (null phenotype). The pattern of mucin phenotype expression was significantly different between group A and group B or C (*Cochran-Armitage* trend test, *p* = 0.005 and *p* = 0.03, respectively); but there were no difference between group B and group C tumours (*p* = 0.642) and between group A/B and C tumours (*p* = 0.147) (Additional file 1: Table S4).

### Quantitative PCR (qPCR) results

In qPCR analyses of 8 randomly selected samples using 5 gene primer sets, the aCGH T/R ratios could not be validated in >50% of the 40 comparisons (data not shown). By comparing the PCR efficiencies between reference samples before and after WGA, we could demonstrate biased amplification; however, this bias was dependent on the examined genes and was reproducible among the samples (Additional file 2: Figure S1).

### Genome-wide CNA patterns

**Figure 4:**
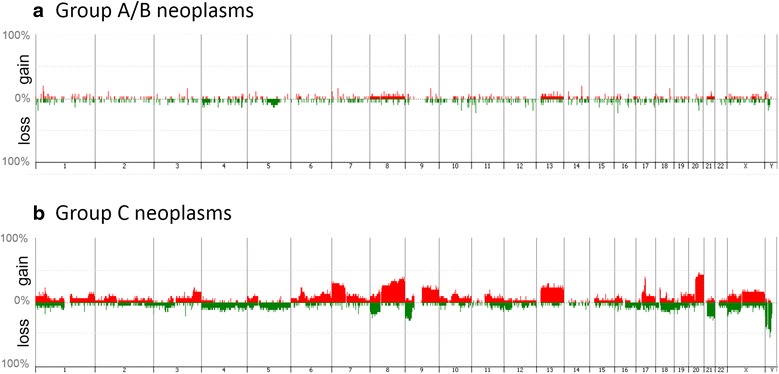
**Frequency of copy-number alterations (CNAs) at the chromosome level.** The percentage of the samples that have CNAs for each chromosome in the group A/B **(a)** and group C **(b)** tumours. Gains and losses are indicated with red and green, respectively.

Penetrance-plots of chromosome-level CNAs are shown for group A/B and group C tumours in Figure 4. Seven group A and 12 group B tumours included 1 and 2 tumours with losses of 5q and 0 and 1 tumour with gain of 8q, respectively, whereas 24 group C tumours included 1 tumour with loss of 5q and 8 tumours with gains of 8q. Gains of 20q were detected in none of group A/B tumours and 9 group C tumours. Chromosome 21q showed gain in 1 group A/B tumour and losses in 5 group C tumours.

To improve the CNA signal-to-noise ratios, the individual probe T/R ratios within a specified gene were averaged. The average T/R ratios of 30,098 gene regions were calculated from 55,023 probes. Based on the average T/R ratio, the significant CNA frequencies in the total analysed gene regions, were 33.0% in group A, 43.6% in group B and 52.2% in group C. The frequencies did not significantly differ between groups A and B (*Welch’s t-*test, *p* = 0.18) or between groups Cm and Cd (*p* = 0.28), but they differed significantly between groups A or B and group C (*p* = 0.02 and *p* = 0.08, respectively).

### Cluster analysis

To compare copy-number profiles between mucosal and deeply invasive parts of individual group Cd tumours, we added data from deeply invasive part samples, of which 3 and 13 were obtained from submucosal and advanced tumours, respectively. The combined data of mucosal and deeply invasive samples were subjected to unsupervised cluster analysis with a gene size-dependent number of genes ranging, from 373 to 9,615 genes. We found that even the 373 genes with 10 or more probes fulfilled the neighbouring standard, except for 1 pair of samples that did not fulfil this standard, irrespective of gene size, and that pair was considered to be derived from a tumour that contained multiple clones (Additional file 3: Figure S2a).

**Figure 5:**
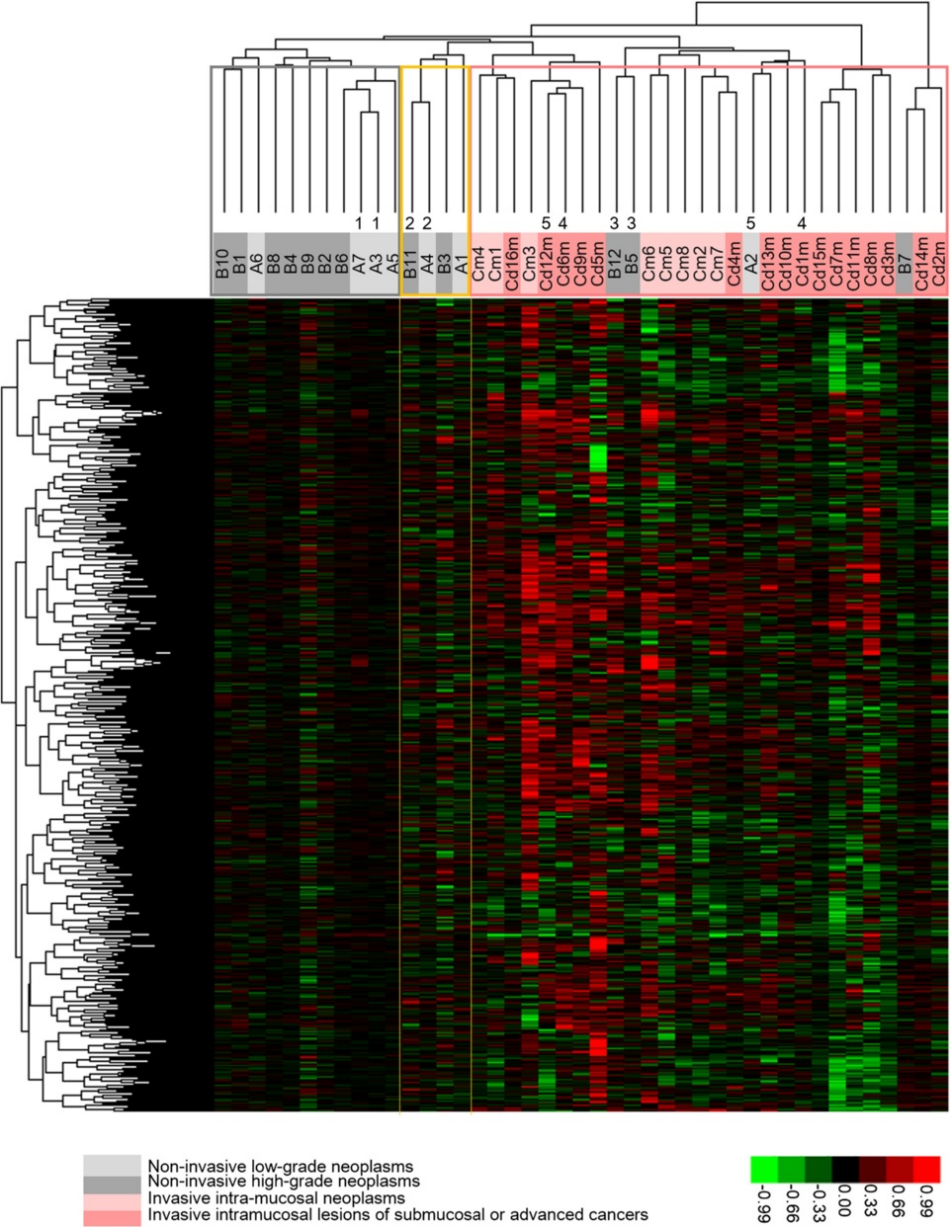
**Unsupervised-hierarchical cluster analysis using 43 samples and 2,863 genes that contain four or more probes.** Genomic copy-number gains or losses are indicated by red and green squares, respectively, for each gene. The length of the heat map is compressed as a 1:60 ratio for visualization. Samples were classified into three clusters: stable, intermediate, and unstable, which are marked with grey, yellow, and pink frames, respectively. The numerals under the dendrogram show five pairs of concurrent tumours. The background colours of the sample names represent morphological categories: light and dark grey indicate non-invasive low-grade and high-grade neoplasms, respectively; light and dark pink indicate invasive intramucosal tumours and intramucosal parts of submucosal or deeper tumours, respectively.

Of the 5 pairs of concomitant but separately distributed lesions in the same patients, under the condition of gene sizes containing up to >6 probes, 3 pairs (A3– A7, B5 –B12 and A4 – B11) were neighbouring to each other in the clustering dendrogram (Figure 5 and Additional file 3: Figure S2b). This genomic copy-number profile similarity between separate lesions in the same patient may be associated with the common carcinogenesis environment but appeared to be less (3 of 5 pairs) than the similarities between different parts of the same lesion (15 of 16 pairs). In each of the neighbouring pairs (B5 –B12 and A4 – B11), one of the pair was labelled with the enzymatic method and the other with the ULS method, showing that there is virtually no difference between the results of the 2 labelling methods.

All 43 tumours were classifiable into 3 major clusters: stable (11 tumours), unstable (28 tumours) and intermediate (4 tumours). As shown in Figure 5, the stable and unstable clusters are characterized by infrequent and frequent copy-number losses/gains, respectively, reflecting different degrees of genetic instability. The intermediate cluster shows intermediate instability. The sample constitution of each was constant under the conditions of the minimal gene size containing 3–4 probes (Additional file 4: Figure S3). Based on these findings, we selected the condition of 2,863 genes containing ≥4 probes (Figure 5). Under this condition, the unstable cluster included all group C tumours and 4 of 19 (21%) of groups A/B tumours. The stable cluster comprised solely of group A/B tumours (4 and 7 in groups A and B, respectively) and accounted for 11 of the 19 (58%) group A/B tumours.

**Figure 6:**
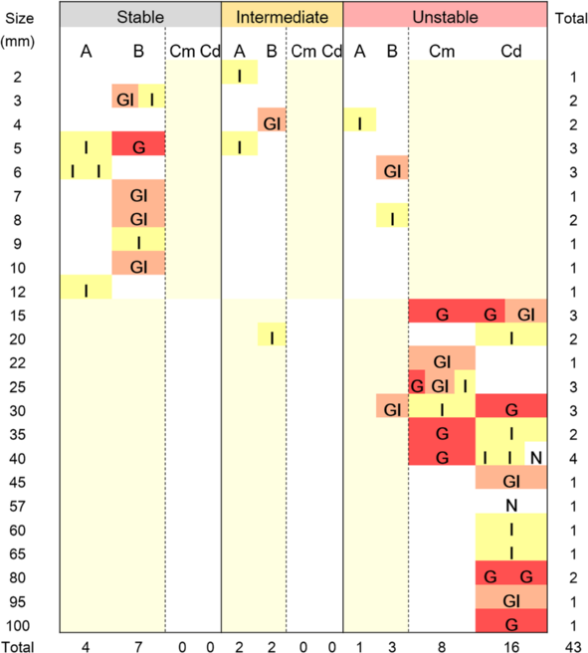
**Relationship of tumour size with mucin phenotypes and histological grades of tumours in stable, intermediate, and unstable clusters.** Each coloured square, representing a sample, was plotted two dimensionally according to increasing tumour size and tumour grade. Light yellow background indicates that samples were not actively collected in these category areas. The numerals on the right side and bottom margins are the total sample number in each line and column, respectively. Regarding G, GI, I and N, see the legend for Figure 3. Regarding Cm and Cd, see the legend for Table 1.

The relationship of tumour size distribution with histological grade and mucin phenotype was demonstrated in each cluster in Figure 6. In the stable cluster, no lesion exceeded 2 cm in diameter. Among the 3 group A/B tumour clusters, the tumour size distribution nearly overlapped, and there was no significant difference in the mean tumour size, suggesting that tumours in these clusters may have occurred in parallel and constitute independent genetic lineages.

For group A/B tumours, the average CNA numbers were significantly greater in the 4 unstable or 4 intermediate tumours than in the 11 stable tumours, with averages of 15,262, 15,727 and 9,370, respectively (*Welch’s t* test, *p* = 0.004 and *p* = 0.006, respectively). The CNA numbers did not differ between the unstable and intermediate clusters (*p* = 0.79). There were no significant differences in the histological grade (Figures 5) or mucin phenotype (Figure 6 and Additional file 1: Table S4) between the type A/B tumours included in the unstable or intermediate clusters and the stable cluster. The unstable/intermediate tumours accounted for 3/7 and 5/12 of the groups A and B tumours, and the intestinal phenotype for 3/7 and 1/12 of the tumours, respectively.

### Genes exhibiting significantly different CNAs among stable, intermediate and unstable clusters

**Figure 7:**
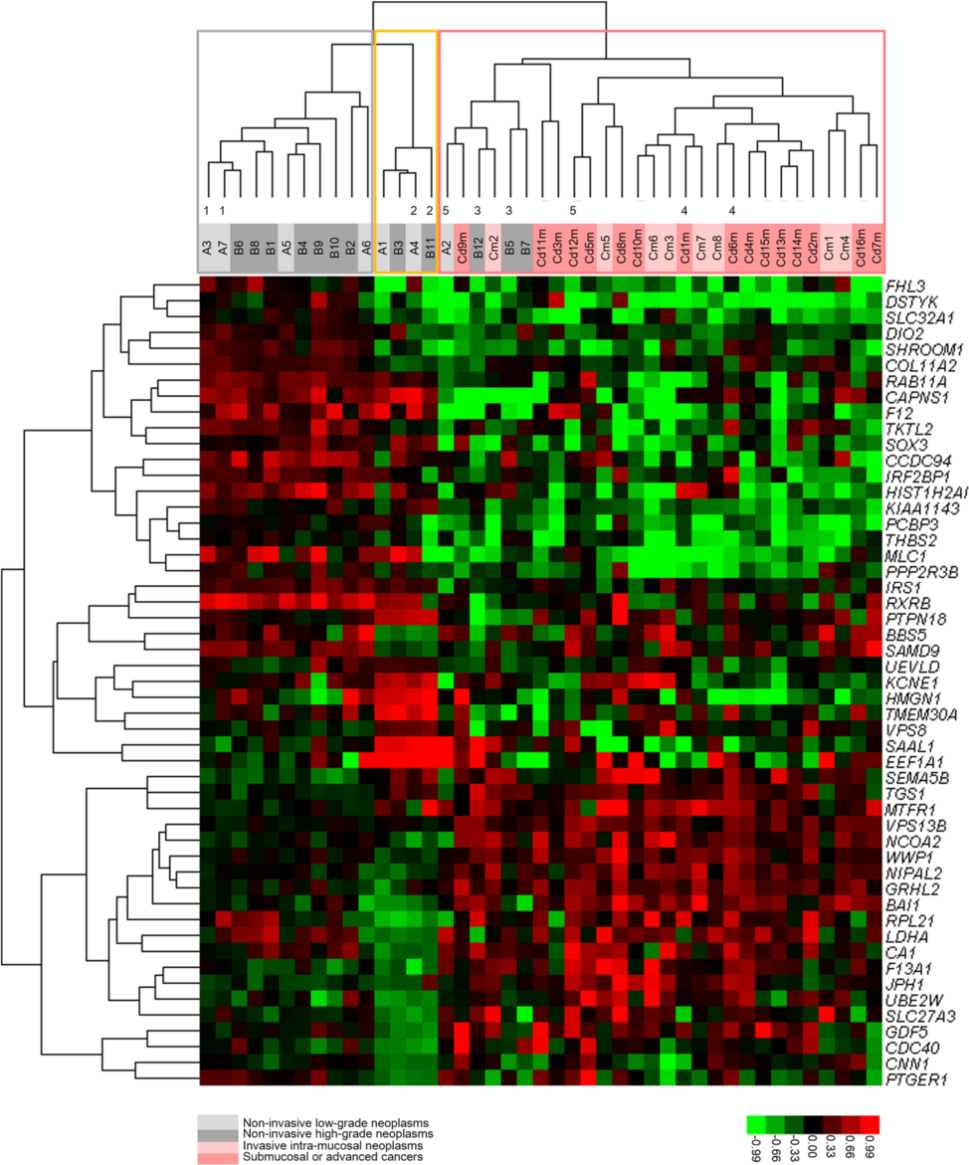
**Two-dimensional supervised cluster analysis using 43 samples and 51 genes.** The cluster showed significantly different copy-numbers from 5 comparisons: stable vs. intermediate, stable vs. unstable, intermediate vs. unstable, stable/intermediate vs. unstable, and stable vs. unstable/intermediate. The statistical data from these comparisons are shown in Additional file 1: Table S5. The clustering results of samples were very similar to those of Figure 5; the samples were classified into 3 clusters: stable, intermediate, and unstable, which are marked with grey, yellow, and pink frames, respectively.

Of the 14,753 protein-coding genes, we identified a total of 51 genes exhibiting significantly different copy-numbers among the stable, intermediate and unstable clusters. Using these genes and 43 tumour samples, we performed two-dimensional hierarchical clustering (Figure 7). A summary of the 51 genes and their significance are shown in Additional file 1: Table S5. The CNAs of these genes in each sample are shown in Additional file 5: Figure S4, in which an association of gene-level CNAs with chromosome-level CNAs was demonstrated. Eleven out of the 51 genes were located in 8q, and often showed gains in the unstable group, associated with the chromosomal gains at 8q (Additional file 5: Figure S4). The 51 genes also contained 2 genes at 5q, 2 genes at 6p, and 2 genes at 21q that showed losses in >50% of the samples in the unstable group, corresponding to chromosome-level losses except in 6p.

## Discussion

In our previous series of small cancers ranging from 0.2 to 2 cm in diameter, the gastric (G/GI) and intestinal (I) types accounted for 88% and 4% of cases, respectively [17], whereas here, in the group B (non-invasive high-grade neoplasia) and Cm (intramucosal invasive carcinoma) tumours, which ranged from 0.3 to 4.0 cm in diameter, the G/GI and I types accounted for 70% and 30% of cases, respectively. This tendency of a shift to I type with tumour size was further enhanced in group Cd (intramucosal part of submucosal or deeper invasive carcinoma) tumours, ranging from 1.5 to 10 cm in diameter, in which the G/GI and I types accounted for 50% and 37.5% of cases, respectively (Figure 3). The decrease in the G/GI-type and coincident increase in the I-type could reflect a loss of the gastric phenotype from G and GI, respectively, secondary to an increase in tumour size, which is consistent with the previously reported notion that a majority of invasive tumours are derived from the gastric lineage [17] and that the gastric phenotype is lost during tumour progression [18,20]. Additionally, there was no significant difference in I-type frequency between the group A/B and group C tumours. These findings indicate that utility of mucin phenotyping may be limited, with respect to outcome predictions at later stages of tumour development, because the lineage markers change during progression. Therefore, in this study we focused on the aCGH approach.

Validation of candidate genes identified using aCGH is critical. However, qPCR is difficult to use for validation purposes because copy-number gains/losses of genomic DNA are too small to detect; a one-copy loss or gain may be equivalent to a 0.5- or 1-cycle difference in qPCR. This may be a reason why we failed to validate more than half of the aCGH T/R ratios here using qPCR. Another reason is the high noise level in the data from DNAs extracted from microdissected FFPE tissues and PCR-amplified. Consequently, we could not comment on the copy-numbers of individual genes as direct results of aCGH. However, we noticed that the amplification bias was reproducible among samples, meaning that the tumour/reference comparison for each microarray spot (probe) and the comparisons of the mean copy-numbers among genes could cancel the amplification bias to some extent. Similarly, copy-number variations (CNVs), present in both tumour and reference samples, were also cancelled.

To validate our aCGH data from another aspect, we compared chromosome-level CNAs detected in this study to those in previous studies. Recent genomic microarray data from gastric cancer have shown that gains of chromosomes 8 and 20 are the most frequent chromosome-level changes [29,30], which was confirmed by our data (Figure 4b). Gains at 8q and losses at 5q were detected in a fraction of non-invasive gastric neoplasms [29], which were reproduced in the present study (Figure 4a), although their frequencies here were lower than those of the previous report partially due to the higher threshold for significant CNA in our study.

At the gene level, we compared the copy-number profiles among the samples by unsupervised hierarchical clustering using average probe copy-numbers. Larger sized genes, for which the representative copy-numbers were determined by averaging a greater number of probe copy-numbers within the gene, were used to cancel out noise in the gene copy-number [27]. The reproducibility of genomic copy-number profiles was also assessed by confirming (1) neighbouring positions in clustering dendrograms of samples from identical tumours [27] after adding samples from deeply invasive parts of the corresponding tumours and (2) consistency in the sample constitution of each cluster during repeated clustering with varying gene sizes. Repeated clustering demonstrated that clustering of the genes that contain ≥4 probes was optimal and that the profiles of 2 separate concurrent lesions in a single patient were less similar than those of different parts of the same lesion.

Clustering under the optimal condition classified 43 intramucosal gland-forming neoplasms of varying histological grades into 3 clusters: stable, unstable and intermediate. The unstable cluster may represent a lineage of poor outcome, consisting of tumours from incipient to advanced stages because this cluster, but not the stable cluster, included invasive carcinomas (Figure 6). There were no significant differences in the histological grades or mucin phenotypes between the 3 clusters. These findings suggest that progression risk may not be primarily related to the histological grade or mucin phenotype but may instead be linked to the lineage-specific, genomic CNA pattern.

Fifty-one genes with significantly different CNAs between the 3 clusters were identified (Additional file 1: Table S5). These CNAs constituted a core profile that could discriminate between the 3 clusters (Figure 7). The 51 genes include the following biologically relevant genes: *RXRB*, a member of the retinoid X receptor family of nuclear receptors, which plays a critical role in the regulation of growth and differentiation in normal and tumour cells [31], *VPS13B*, which is mutated in gastric and colorectal cancers, and in Cohen syndrome with high microsatellite instability [32] and is coamplified with *MYC* in breast cancers [33], and *NCOA2*, encodes nuclear receptor coactivator 2, related to the function of nuclear hormone receptors and amplified or overexpressed in prostate cancers [34]. As shown in Additional file 5: Figure S4, CNAs of *RXRB* were found mostly as gains in the stable and intermediate groups (14/15), but were often found as losses or unchanged in the unstable group (24/28), consistent with the tumour-suppressing functions of *RXRB*. Copy-numbers of *VPS13B* and *NCOA2* were mostly unchanged in the stable and intermediate groups (15/15 and 14/15, respectively), but were frequently gained in the unstable group (15/28 and 19/28, respectively), consistent with the oncogenic functions of these genes. Not only these 2 genes but another 9 out of the 51 were located in 8q, and they often showed gains in the unstable group (Additional file 5: Figure S4), which may be related to the 8q gain at the chromosome level. At least 12 of the 51 genes showed CNAs that were parallel to the corresponding chromosome-level CNAs, which were reported previously [29,30]. These non-random gene-level CNAs, which are difficult to explain as noises or artefacts, are more sensitively associated with invasive (group C) tumours than are infrequent chromosome-level changes. The generating mechanism of CNAs, unrelated to chromosomal changes, remains to be explained.

**Figure 8:**
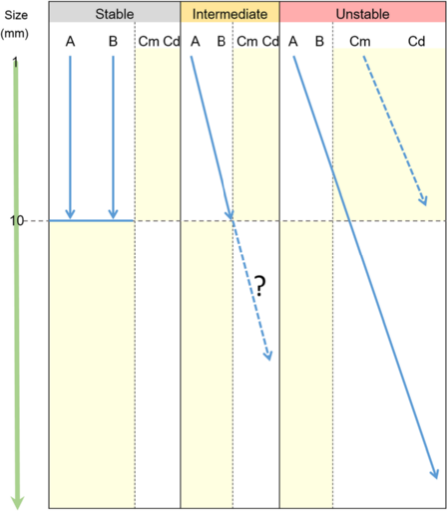
**A schematic chart of 3 lineages in the development of gland-forming gastric neoplasms.** This figure is based on Figure 6 and similarly shows the size distribution of 3 lineages: stable, intermediate and unstable. These lineages may be determined by the profile of genomic CNAs in the incipient phase of tumour growth. The growth of stable tumours may be limited in the mucosa and plateau around the size of 1 cm, whereas the unstable tumours may largely occur as *de-novo* carcinomas and partly derive from intraepithelial neoplasia, accumulate genetic changes and become intramucosal and deeply invasive tumours. The dotted line in unstable lineage indicates small *de-novo* carcinomas, which were not examined in the present study. The dotted line in intermediate lineage shows that the outcome of this lineage remains to be clarified. Regarding Cm and Cd, see the legend for Table 1.

Non-invasive neoplasms of intermediate and unstable lineage had higher levels of genomic instability than stable-lineage neoplasms (Figure 5). In the stable lineage, there was no significant size difference between LGNs and HGNs, whereas in the unstable and intermediate lineages, LGNs tended to be smaller in size than HGNs (Figure 6). There was a pair of concurrent LGN and HGN, which were located in the neighbouring positions in the intermediate cluster, indicating that these tumours can share highly similar CNAs despite differences in the histological grade. These findings suggest that a fraction of smaller LGNs can change to HGNs during tumour development as demonstrated by follow-up studies [6-15]. In addition, larger non-invasive neoplasms are more frequently found to be HGNs, which may explain why HGNs have a higher risk of progression to invasive carcinomas compared with LGNs (Figure 8). It remains to be determined if non-invasive intermediate-lineage neoplasms can progress to invasive carcinoma and if small carcinomas arising *de novo* actually show the unstable CNA profile. More cases should be examined to clarify these points.

It is unlikely that tumours in the stable cluster stochastically accumulate CNAs to become unstable tumours because opposing CNAs were detected among the 3 lineages (Figure 7). Additionally, cell kinetic studies have indicated that approximately two-thirds to three-quarters of the natural history of GC has already elapsed in intramucosal tumours of 1 cm in diameter [35]. This means that, even in small non-invasive tumours, there is sufficient time to accumulate genomic changes that determine the potential of tumour aggressiveness, leaving limited opportunity for further CNA accumulation.

In the near future, development of custom microarrays for determination of the copy-numbers of essential genes we extracted may enable us to apply our study to clinical practice. By applying our approach to endoscopically removed mucosal lesions, the necessity of additional surgical treatment can be determined. This may give patents to chance for early treatment of high-risk lesions and relieve patients with low-risk lesions from unnecessary surgical excision of stomach.

## Conclusions

Relative comparisons of genomic copy-number profiles by unsupervised hierarchical clustering enabled us to categorize gastric intramucosal neoplasms according to lineage-specific patterns of CNAs and different degrees of genomic copy-number instability as stable, unstable, and intermediate. Since invasive carcinomas were included only in the unstable cluster, non-invasive neoplasms of the unstable cluster, accounting for 21% of non-invasive neoplasms, may accumulate genetic changes in a stochastic manner and progress to invasive GCs, whereas those of the stable cluster, accounting for 58% of non-invasive neoplasms may not. This classification was not significantly correlated with the histological grade, mucin phenotype or size of non-invasive tumours, but was consistent with the results of previous long-term follow-up studies.

### Availability and requirements

The microarray data were registered in the GEO database (URL: http://www.ncbi.nlm.nih.gov/geo/query/acc.cgi?acc=GSE60116).

## Additional files

Additional file 1: Table S1. Primer sequences used in quantitative PCR. **Table S2a and b.** Clinicopathological features of groups A, B and C. **Table S3.** Mucin phenotypic expressions in groups A, B, C. **Table S4.** Constitution of phenotypic expression in groups (A, B and C) and clusters (*stable*, *intermediate* and *unstable*). **Table S5.** List of 51 genes extracted by *t* test with *Bonferroni correction* from 5 different comparisons. Additional file 2: Figure S1. Plot of PCR cycle number, at which the PCR products exceed the threshold (Cq). The Cq value profiles of 5 genes are shown for 8 reference samples before **(a)** and after **(b)** whole genome amplification. The genes used are markers of chromosome 15 and 10 (#51481794-51481853 and # 2681585–72681644, respectively), *MYC*, *TP53*, and *MME*. Additional file 3: Figure S2. Two-dimensional supervised cluster analysis using 43 mucosal and 16 invasive–part samples. To compare the copy-number profile of mucosal and invasive parts of individual tumours in the clustering analyses with varying size-dependent gene numbers, 16 samples from invasive parts of Cd tumours were added to the 43 mucosal samples. Following “Cd”, the sample numbers with and without “m” indicate intramucosal and extramucosal parts, respectively. **(a)** Clustering dendrograms with varying gene numbers, from 9,615 genes containing ≥2 probes to 373 genes containing ≥10 probes. Thick red frames are pairs of mucosal and invasive samples from identical tumours. These pairs are consistently neighbouring except for 1 pair of samples marked with closed red arrows. Of the pairs of concurrent tumours that were located separately from each other in the single patient and marked with a pair of numbers under the sample name, the pairs that are located in neighbouring positions in the dendrogram are marked with thin red frames; those in split positions are marked with pairs of closed or open black arrows. **(b)** A heat map of the clustering that corresponds to the upper right dendrogram of (a), using 2,863 genes of ≥4 probes. Grey and yellow squares indicate the samples of the stable and the intermediate clusters, respectively. The samples without a frame belong to the unstable cluster. Additional file 4: Figure S3. Two-dimensional supervised cluster analysis using 43 samples. In this series, sample constitutions of the clusters were stable under the condition of gene size, containing ≥3-4 probes. The grey, yellow, and red underbars indicate stable intermediate and unstable clusters, respectively, as defined under this optimal condition. Additional file 5: Figure S4. Tumour/reference (T/R) ratios of the 51 candidate genes in the samples of 3 clusters. The T/R ratio of each gene represents the average T/R ratios of probes within the gene. Significant copy-number gains and losses are defined as >0.3219 and <−0.3219, and are marked with pink, and green boxes, respectively. Black frames indicate the genes mentioned in the text: *RXRB*, *VPS13B*, and *NCOA2*. Regarding the background colour of “Location” column, pink and green indicate gains and losses at the chromosome arm level (shown in Figure 4), respectively. In the column labelled “Association of chromosomal CNAs with gene CNAs”, positive associations of chromosomal arm-level CNAs indicated by the colour of the “Location” column with gene-level CNAs in >50% of samples of the stable, intermediate, or unstable group are marked with S, I, and U, respectively.
