# Supplementary material for: Progression risk assessments of individual non-invasive gastric neoplasms by genomic copy-number profile and mucin phenotype
Source: BMC Med Genomics. 2015 Feb 18;8:6. doi: 10.1186/s12920-015-0080-6 (PMC4346124; doi:10.1186/s12920-015-0080-6)
Supplement: Additional file 1: Table S1. — Primer sequences used in quantitative PCR. Table S2a and b. Clinicopathological features of groups A, B and C. Table S3. Mucin phenotypic expressions in groups A, B, C. Table S4. Constitution of phenotypic expression in groups (A, B and C) and clusters (stable, intermediate and unstable). Table S5. List of 51 genes extracted by t test with Bonferroni correction from 5 different comparisons. [file 12920_2015_80_MOESM1_ESM.docx]

# Additional files

## Table S1 - Primer sequences used in quantitative PCR

| **Primer** | **Sequence** |
| --- | --- |
| chr15* Forward | AGTTTCGTTCTATACTTCACCTTGG |
| chr15* Reverse | TCATCATTATTATTTTCCCATGACC |
| chr10** Forward | TATGCATGGGTGATGTGCTT |
| chr10** Reverse | CAGAGGGAACCCTACATTCG |
| *MYC* Forward | ATCCTTTTAAGAAGTTGGCATTTG |
| *MYC* Reverse | CACGTCCTAACACCTCTAGAGACC |
| *TP53* Forward | CTGAGGAGTGTCCGAAGAGAAT |
| *TP53* Reverse | ACTGCAAAGCTTCTGGAAGAAC |
| *MME* Forward | GGACTTAACCCTGTGTCTTTGG |
| *MME* Reverse | CCTGAAATACGGAGTTCTGGAC |

*chr15:51481794-51481853

**chr10:72681585-72681644

## Table S2a - Clinicopathological features of groups A, B, and C

| **No** | **Case  No** | **Age** | **Sex** | **Size (mm)** | **Macroscopic type*** | **Vienna classification** | **Resection method** |
| --- | --- | --- | --- | --- | --- | --- | --- |
| 1 | A1 | 81 | M | 2 | 0-IIb | 3 | ESD |
| 2 | A2^(5)^ | 78 | M | 4 | 0-I | 3 | OP |
| 3 | A3^(1)^ | 59 | F | 5 | 0-I | 3 | ESD |
| 4 | A4^(2)^ | 66 | M | 5 | 0-IIa | 3 | OP |
| 5 | A5 | 61 | M | 6 | 0-I | 3 | ESD |
| 6 | A6 | 65 | F | 6 | 0-IIa | 3 | ESD |
| 7 | A7^(1)^ | 59 | F | 12 | 0-I | 3 | ESD |
| 8 | B1 | 41 | M | 3 | 0-IIb | 4 | ESD |
| 9 | B2 | 67 | M | 3 | 0-IIc | 4 | ESD |
| 10 | B3 | 67 | F | 4 | 0-IIb | 4 | ESD |
| 11 | B4 | 60 | M | 5 | 0-IIc | 4 | ESD |
| 12 | B5^(3)^ | 72 | F | 6 | 0-IIa | 4 | OP |
| 13 | B6 | 52 | F | 7 | 0-IIb | 4 | ESD |
| 14 | B7 | 61 | M | 8 | 0-IIc | 4 | ESD |
| 15 | B8 | 59 | M | 8 | 0-I | 4 | OP |
| 16 | B9 | 77 | F | 9 | 0-I | 4 | ESD |
| 17 | B10 | 61 | M | 10 | 0-IIb | 4 | ESD |
| 18 | B11^(2)^ | 66 | M | 20 | 0-IIa | 4 | OP |
| 19 | B12^(3)^ | 72 | F | 30 | 0-IIc | 4 | OP |

*Histology type, macroscopic type, depth of invasion, pTNM staging is according to the Japanese gastric cancer classification [23]. OP: surgical resection; ESD: endoscopic submucosal dissection. Concurrent lesions are noted as superscript from (1) to (5) attached to case number.

## Table S2b - (cont.)

| **No** | **Sample** | **Age** | **Sex** | **Size (mm)** | **Histological type*** | **Depth of invasion*** | **pT** | **pN** | **pM** | **ly** | **v** | **Stage** |
| --- | --- | --- | --- | --- | --- | --- | --- | --- | --- | --- | --- | --- |
| 1 | Cm1 | 73 | M | 15 | tub | M | 1a | 0 | 0 |  |  | 1A |
| 2 | Cm2 | 76 | M | 22 | pap>tub2 | M | 1a | 0 | 0 |  |  | 1A |
| 3 | Cm3 | 59 | M | 25 | pap>tub | M | 1a | 0 | 0 |  |  | 1A |
| 4 | Cm4 | 80 | F | 25 | tub2>por | M | 1a | 0 | 0 |  |  | 1A |
| 5 | Cm5 | 68 | M | 25 | tub | M | 1a | 0 | 0 |  |  | 1A |
| 6 | Cm6 | 81 | M | 30 | tub | M | 1a | 0 | 0 |  |  | 1A |
| 7 | Cm7 | 65 | M | 35 | tub>pap | M | 1a | 0 | 0 |  |  | 1A |
| 8 | Cm8 | 92 | F | 40 | pap>tub | M | 1a | 0 | 0 |  |  | 1A |
| 9 | Cd1m^(4)^ | 82 | M | 15 | tub | MP | 2 | 1 | 0 |  |  | IIA |
| 10 | Cd2m | 64 | F | 15 | tub2>pap | SM1b | 1b | 1 | 0 | + | + | IB |
| 11 | Cd3m | 75 | F | 20 | tub2>por2 | SM1b | 1b | 1 | 0 | + |  | IB |
| 12 | Cd4m | 70 | M | 30 | tub | MP | 2 | 0 | 0 | + |  | IB |
| 13 | Cd5m | 88 | F | 35 | pap+tub | MP | 2 | 0 | 0 |  |  | IB |
| 14 | Cd6m^(4)^ | 82 | M | 40 | tub2 | SS | 3 | 1 | 0 | + | + | IIB |
| 15 | Cd7m | 72 | M | 40 | tub2>pap | SS | 3 | 0 | 0 | + | + | IIA |
| 16 | Cd8m | 65 | M | 40 | tub2>por2 | SS | 3 | 3b | 0 | + | + | IIIB |
| 17 | Cd9m | 79 | M | 45 | tub>por1 | SE | 4a | 2 | 0 | + | + | IIIB |
| 18 | Cd10m | 83 | M | 57 | tub >pap | SM1b | 1b | 0 | 0 |  |  | IA |
| 19 | Cd11m | 85 | M | 60 | tub>pap | SS | 3 | 3a | 0 | + | + | IIIB |
| 20 | Cd12m^(5)^ | 78 | M | 65 | tub2 | SS | 3 | 2 | 0 |  | + | IIIA |
| 21 | Cd13m | 80 | M | 80 | tub>por2+pap | SS | 3 | 3a | 0 | + |  | IIIB |
| 22 | Cd14m | 79 | F | 80 | tub2>por | SS | 3 | 3b | 0 | + | + | IIIB |
| 23 | Cd15m | 71 | M | 95 | tub | SE | 4a | 3a | 0 | + | + | IIIC |
| 24 | Cd16m | 86 | M | 100 | tub>pap | SS | 3 | 1 | 0 | + |  | IIB |

## Table S3 - Mucin phenotypic expressions in groups A, B, and C

| **No** | **Case No** | **MUC5AC** | **MUC6** | **MUC2** | **CD10** | **Phenotype** |
| --- | --- | --- | --- | --- | --- | --- |
| 1 | A1 | n | n | - | 3+ | I |
| 2 | A2^(5)^ | n | n | 1+ | 2+ | I |
| 3 | A3^(1)^ | n | n | 1+ | 3+ | I |
| 4 | A4^(2)^ | n | n | 3+ | 2+ | I |
| 5 | A5 | n | - | - | 3+ | I |
| 6 | A6 | n | n | 1+ | 1+ | I |
| 7 | A7^(1)^ | n | n | 3+ | 3+ | I |
| 8 | B1 | - | - | 1+ | 2+ | I |
| 9 | B2 | 1+ | - | - | 3+ | G<I |
| 10 | B3 | - | 1+ | 1+ | 1+ | G<I |
| 11 | B4 | 3+ | 2+ | - | n | G |
| 12 | B5^(3)^ | - | 1+ | 2+ | 3+ | G<I |
| 13 | B6 | 1+ | 1+ | - | 2+ | G=I |
| 14 | B7 | n | - | 1+ | 3+ | I |
| 15 | B8 | - | 1+ | 1+ | 2+ | G<I |
| 16 | B9 | - | - | 2+ | 2+ | I |
| 17 | B10 | 1+ | - | 1+ | - | G=I |
| 18 | B11^(2)^ | - | - | 1+ | 3+ | I |
| 19 | B12^(3)^ | - | 1+ | 1+ | 3+ | G<I |
| 20 | Cm1 | 2+ | 3+ | - | - | G |
| 21 | Cm2 | 1+ | 1+ | 1+ | - | G>I |
| 22 | Cm3 | 2+ | 2+ | 1+ | 2+ | G>I |
| 23 | Cm4 | 3+ | 1+ | - | - | G |
| 24 | Cm5 | - | - | 1+ | 3+ | I |
| 25 | Cm6 | - | - | 1+ | 2+ | I |
| 26 | Cm7 | 3+ | 2+ | - | - | G |
| 27 | Cm8 | 3+ | 1+ | - | - | G |
| 28 | Cd1m^(4)^ | 1+ | 1+ | - | - | G |
| 29 | Cd2m | 2+ | 1+ | 1+ | - | G>I |
| 30 | Cd3m | - | - | 1+ | - | I |
| 31 | Cd4m | 1+ | - | n | - | G |
| 32 | Cd5m | - | - | n | 1+ | I |
| 33 | Cd6m^(4)^ | - | - | - | - | N |
| 34 | Cd7m | - | n | n | 3+ | I |
| 35 | Cd8m | n | - | 1+ | n | I |
| 36 | Cd9m | - | 1+ | - | 3+ | G<I |
| 37 | Cd10m | - | - | - | - | N |
| 38 | Cd11m | - | - | 1+ | - | I |
| 39 | Cd12m^(5)^ | - | - | 1+ | 3+ | I |
| 40 | Cd13m | 1+ | 1+ | n | n | G |
| 41 | Cd14m | 3+ | 3+ | n | n | G |
| 42 | Cd15m | - | 1+ | 2+ | 1+ | G<I |
| 43 | Cd16m | 3+ | 2+ | n | - | G |

## Table S4 - Constitution of phenotypic expression in groups (A, B, and C) and clusters (*stable*, *intermediate,* and *unstable*)

|  | | **Phenotypic expression** | | | |
| --- | --- | --- | --- | --- | --- |
|  |  | **G** | **GI** | **I** | **N** |
| Group | A | 0 | 0 | 7 | 0 |
|  | B | 1 | 7 | 4 | 0 |
|  | Cm | 4 | 2 | 2 | 0 |
|  | Cd | 5 | 3 | 6 | 2 |
| Comparison (Cochran Armitage trend test) | A vs B | 0.005* | | | |
|  | A vs C | 0.030* | | | |
|  | Cm vs Cd | 0.211 | | | |
|  | B vs Cm | 0.149 | | | |
|  | B vs Cd | 0.842 | | | |
|  | A/B vs C | 0.147 | | | |
| Cluster | Stable (S) | 1 | 4 | 6 | 0 |
|  | Intermediate (I) | 0 | 1 | 3 | 0 |
|  | Unstable (U) | 9 | 7 | 10 | 2 |
| Comparison (Cochran Armitage trend test) | S vs I | 0.375 | | | |
|  | I vs U | 0.233 | | | |
|  | S vs U | 0.298 | | | |

*Significant differences are considered as bilateral *p* value ≤0.05

## Table S5 - List of 51 genes extracted by *t* test with *Bonferroni correction* from 5 different comparisons

| **No** | **Gene symbol** | **Gene name** | **HUGO gene nonmenclature ID** | **Location** | **Identified probe** | **Comparison** | ***t* test** | |
| --- | --- | --- | --- | --- | --- | --- | --- | --- |
|  |  |  |  |  |  |  | ***p value*** | ***p* value after  Bonferroni correction** |
| 1 | *FHL3* | four and a half LIM domains 3 | HGNC:3704 | 1p34.3 | 1 | 11 S vs 32 IU | 9.82838E-07 | 0.014499814 |
|  |  |  |  |  |  | 11 S vs 28U | 2.45323E-06 | 0.036192457 |
| 2 | *DSTYK* | dual serine/threonine and tyrosine protein kinase | HGNC:29043 | 1q32 | 1 | 11 S vs 32 IU | 1.35217E-06 | 0.019948632 |
| 3 | *SLC32A1* | solute carrier family 32 (GABA vesicular transporter), member 1 | HGNC:11018 | 20q11 | 1 | 11 S vs 32 IU | 4.47098E-08 | 0.000659604 |
|  |  |  |  |  |  | 11 S vs 28 U | 2.06923E-07 | 0.003052734 |
| 4 | *DIO2* | deiodinase, iodothyronine, type II | HGNC:2884 | 14q24.2-q24.3 | 4 | 11 S vs 32 IU | 2.05526E-07 | 0.003032126 |
|  |  |  |  |  |  | 11 S vs 28 U | 8.07541E-08 | 0.001191365 |
| 5 | *SHROOM1* | shroom family member 1 | HGNC:24084 | 5q31.1 | 1 | 11 S vs 32 IU | 1.09434E-08 | 0.000161448 |
|  |  |  |  |  |  | 11 S vs 28 U | 1.0981E-07 | 0.001620029 |
| 6 | *COL11A2* | collagen, type XI, alpha 2 | HGNC:2187 | 6p21.3 | 3 | 11 S vs 32 IU | 6.27525E-07 | 0.009257877 |
| 7 | *RAB11A* | RAB11A, member RAS oncogene family | HGNC:9760 | 15q22.31 | 1 | 11 S vs 32 U | 1.16426E-06 | 0.017176308 |
|  |  |  |  |  |  | 15 SI vs 28 U | 2.70238E-06 | 0.039868251 |
|  |  |  |  |  |  | 11 S vs 28 U | 1.10129E-06 | 0.01624736 |
| 8 | *CAPNS1* | calpain, small subunit 1 | HGNC:1481 | 19q13.1 | 1 | 15 SI vs 28 U | 1.42563E-06 | 0.021032346 |
| 9 | *F12* | coagulation factor XII (Hageman factor) | HGNC:3530 | 5q35.3 | 1 | 15 SI vs 28 U | 3.263E-07 | 0.0048139 |
|  |  |  |  |  |  | 11 S vs 28 U | 1.0769E-06 | 0.015887463 |
| 10 | *TKTL2* | transketolase-like 2 | HGNC:25313 | 4q32.2 | 1 | 11 S vs 32 IU | 2.31316E-06 | 0.03412603 |
| 11 | *SOX3* | SRY (sex determining region Y)-box 3 | HGNC:11199 | Xq27.1 | 2 | 15 SI vs 28 U | 2.69489E-07 | 0.003975777 |
|  |  |  |  |  |  | 11 S vs 28 U | 6.50137E-07 | 0.009591465 |
| 12 | *CCDC94* | coiled-coil domain containing 94 | HGNC:25518 | 19p13.3 | 2 | 11 S vs 32 IU | 8.54309E-07 | 0.012603618 |
|  |  |  |  |  |  | 11 S vs 28 U | 1.06791E-06 | 0.015754844 |
| 13 | *IRF2BP1* | interferon regulatory factor 2 binding protein 1 | HGNC:21728 | 19q13.32 | 2 | 11 S vs 32 IU | 2.93674E-06 | 0.043325674 |
| 14 | *HIST1H2AI* | histone cluster 1, H2ai | HGNC:4725 | 6p22.1 | 1 | 11 S vs 32 IU | 3.50554E-07 | 0.005171728 |
|  |  |  |  |  |  | 11 S vs 28 U | 2.66901E-07 | 0.003937586 |
| 15 | *KIAA1143* | *KIAA1143* | HGNC:29198 | 3p21.31 | 2 | 11 S vs 32 IU | 3.21833E-06 | 0.047480032 |
|  |  |  |  |  |  | 15 SI vs 28 U | 8.24555E-08 | 0.001216466 |
|  |  |  |  |  |  | 11 S vs 28 U | 1.26514E-07 | 0.001866466 |
| 16 | *PCBP3* | poly(rC) binding protein 3 | HGNC:8651 | 21q22.3 | 2 | 11 S vs 32 IU | 1.90924E-08 | 0.00028167 |
|  |  |  |  |  |  | 11 S vs 28 U | 7.81468E-09 | 0.00011529 |
| 17 | *THBS2* | thrombospondin 2 | HGNC:11786 | 6q27 | 1 | 11 S vs 32 IU | 2.46703E-07 | 0.003639611 |
|  |  |  |  |  |  | 11 S vs 28 U | 6.93897E-07 | 0.010237066 |
| 18 | *MLC1* | megalencephalic leukoencephalopathy with subcortical cysts 1 | HGNC:17082 | 22q13.33 | 1 | 11 S vs 28 U | 3.25292E-06 | 0.047990312 |
| 19 | *PPP2R3B* | protein phosphatase 2, regulatory subunit B'', beta | HGNC:13417 | Xp22.3 and Yp11.3 | 3 | 11 S vs 28 U | 3.33289E-06 | 0.04917013 |
| 20 | *IRS1* | insulin receptor substrate 1 | HGNC:6125 | 2q36 | 2 | 11 S vs 32 IU | 3.09264E-06 | 0.045625778 |
|  |  |  |  |  |  | 15 SI vs 28 U | 3.17104E-07 | 0.004678235 |
|  |  |  |  |  |  | 11 S vs 28 U | 9.09683E-07 | 0.01342055 |
| 21 | *RXRB* | retinoid X receptor, beta | HGNC:10478 | 6p21.3 | 1 | 11 S vs 32 IU | 5.02328E-09 | 7.41085E-05 |
|  |  |  |  |  |  | 15 SI vs 28 U | 1.60973E-06 | 0.023748377 |
|  |  |  |  |  |  | 11 S vs 28 U | 2.57181E-09 | 3.79418E-05 |
| 22 | *PTPN18* | protein tyrosine phosphatase, non-receptor type 18 (brain-derived) | HGNC:9649 | 2q21 | 2 | 11 S vs 4 I | 1.09025E-08 | 0.000160844 |
| 23 | *BBS5* | Bardet-Biedl syndrome 5 | HGNC:970 | 2q31 | 2 | 4 I vs 28 U | 2.46458E-07 | 0.00363599 |
| 24 | *SAMD9* | sterile alpha motif domain containing 9 | HGNC:1348 | 7q21.2 | 1 | 11 S vs 4 I | 1.5483E-06 | 0.022842109 |
| 25 | *UEVLD* | UEV and lactate/malate dehyrogenase domains | HGNC:30866 | 11p15.1 | 4 | 4 I vs 28 U | 1.05191E-07 | 0.001551882 |
| 26 | *KCNE1* | potassium channel, voltage-gated, , Isk-related subfamily, member 1 | HGNC:6240 | 21q22.1-q22.2 | 2 | 4 I vs 28 U | 1.31155E-06 | 0.019349264 |
| 27 | *HMGN1* | high mobility group nucleosome binding domain 1 | HGNC:4984 | 21q22.3 | 1 | 4 I vs 28 U | 2.92559E-08 | 0.000431613 |
| 28 | *TMEM30A* | transmembrane protein 30A | HGNC:16667 | 6q14.1 | 2 | 4 I vs 28 U | 3.44255E-08 | 0.000507879 |
| 29 | *VPS8* | vacuolar protein sorting 8 homolog (S. cerevisiae) | HGNC:29122 | 3q27.2 | 5 | 11 S vs 4 I | 1.78442E-06 | 0.026325478 |
| 30 | *SAAL1* | serum amyloid A-like 1 | HGNC:25158 | 11p15.1 | 1 | 11 S vs 4 I | 3.33267E-07 | 0.00491669 |
| 31 | *EEF1A1* | eukaryotic translation elongation factor 1 alpha 1 | HGNC:3189 | 6q14.1 | 1 | 11 S vs 4 I | 8.37703E-07 | 0.012358636 |
| 32 | *SEMA5B* | sema domain, seven thrombospondin repeats (type 1 and type 1-like), transmembrane domain (TM) and short cytoplasmic domain, (semaphorin) 5B | HGNC:10737 | 3q21.1 | 2 | 11 S vs 32 IU | 4.43282E-07 | 0.006539733 |
|  |  |  |  |  |  | 11 S vs 28 U | 2.28392E-06 | 0.033694689 |
| 33 | *TGS1* | trimethylguanosine synthase 1 | HGNC:17843 | 8q11 | 2 | 11 S vs 32 IU | 1.71005E-06 | 0.025228379 |
| 34 | *MTFR1* | mitochondrial fission regulator 1 | HGNC:29510 | 8q13.1 | 3 | 11 S vs 32 IU | 6.32177E-07 | 0.009326501 |
|  |  |  |  |  |  | 11 S vs 28 U | 8.79864E-07 | 0.012980631 |
| 35 | *VPS13B* | vacuolar protein sorting 13 homolog B (yeast) | HGNC:2183 | 8q22-q23 | 18 | 11 S vs 32 IU | 1.29566E-07 | 0.001911493 |
|  |  |  |  |  |  | 15 SI vs 28 U | 6.516E-09 | 9.61305E-05 |
|  |  |  |  |  |  | 11 S vs 28 U | 1.67141E-08 | 0.000246583 |
| 36 | *NCOA2* | nuclear receptor coactivator 2 | HGNC:7669 | 8q13.3 | 7 | 15 SI vs 28 U | 2.82575E-06 | 0.041688263 |
| 37 | *WWP1* | WW domain containing E3 ubiquitin protein ligase 1 | HGNC:17004 | 8q21.3 | 2 | 15 SI vs 28 U | 5.84908E-07 | 0.008629148 |
| 38 | *NIPAL2* | NIPA-like domain containing 2 | HGNC:25854 | 8q22.2 | 5 | 15 SI vs 28 U | 9.51024E-07 | 0.014030464 |
| 39 | *GRHL2* | grainyhead-like 2 (Drosophila) | HGNC:2799 | 8q22.3 | 5 | 15 SI vs 28 U | 2.44677E-06 | 0.03609713 |
|  |  |  |  |  |  | 11 S vs 28 U | 3.28526E-06 | 0.048467374 |
| 40 | *BAI1* | brain-specific angiogenesis inhibitor 1 | HGNC:943 | 8q24.3 | 3 | 4 I vs 28 U | 9.65658E-07 | 0.014246358 |
| 41 | *RPL21* | ribosomal protein L21 | HGNC:10313 | 13q12.2 | 2 | 4 I vs 28 U | 7.8571E-07 | 0.011591579 |
| 42 | *LDHA* | lactate dehydrogenase A | HGNC:6535 | 11p15.1 | 1 | 4 I vs 28 U | 2.1244E-09 | 3.13413E-05 |
| 43 | *CA1* | carbonic anhydrase I | HGNC:1368 | 8q21.2 | 2 | 4 I vs 28 U | 5.18977E-07 | 0.007656464 |
| 44 | *F13A1* | coagulation factor XIII, A1 polypeptide | HGNC:3531 | 6p24.2-p23 | 3 | 15 SI vs 28 U | 3.00863E-06 | 0.044386261 |
| 45 | *JPH1* | junctophilin 1 | HGNC:14201 | 8q21 | 4 | 11 S vs 28 U | 1.02881E-06 | 0.015178074 |
| 46 | *UBE2W* | ubiquitin-conjugating enzyme E2W (putative) | HGNC:25616 | 8q21.11 | 2 | 4 I vs 28 U | 6.83879E-09 | 0.000100893 |
| 47 | *SLC27A3* | solute carrier family 27 (fatty acid transporter), member 3 | HGNC:10997 | 1q21.1 | 1 | 4 I vs 28 U | 2.67077E-06 | 0.039401925 |
| 48 | *GDF5* | growth differentiation factor 5 | HGNC:4220 | 20q11.2 | 1 | 4 I vs 28 U | 3.75915E-07 | 0.005545881 |
| 49 | *CDC40* | cell division cycle 40 | HGNC:17350 | 6q22.1 | 1 | 4 I vs 28 U | 2.40453E-07 | 0.003547409 |
| 50 | *CNN1* | calponin 1, basic, smooth muscle | HGNC:2155 | 19p13.2-p13.1 | 1 | 11 S vs 4 I | 2.69634E-06 | 0.039779062 |
| 51 | *PTGER1* | prostaglandin E receptor 1 (subtype EP1), 42kDa | HGNC:9593 | 19p13.1 | 1 | 11 S vs 4 I | 2.29431E-06 | 0.03384792 |

In the column of “Comparison”, 11 S, 28 U, 4 I, 15 SI, and 32 IU indicate 11 samples of the stable cluster, 28 samples of the unstable cluster, 4 samples of the intermediate cluster, 15 samples of combined stable and intermediate clusters, and 32 samples of combined intermediate and unstable clusters, respectively.
