# Supplementary figures and images for: Progression risk assessments of individual non-invasive gastric neoplasms by genomic copy-number profile and mucin phenotype
Source: BMC Med Genomics. 2015 Feb 18;8:6. doi: 10.1186/s12920-015-0080-6 (PMC4346124; doi:10.1186/s12920-015-0080-6)

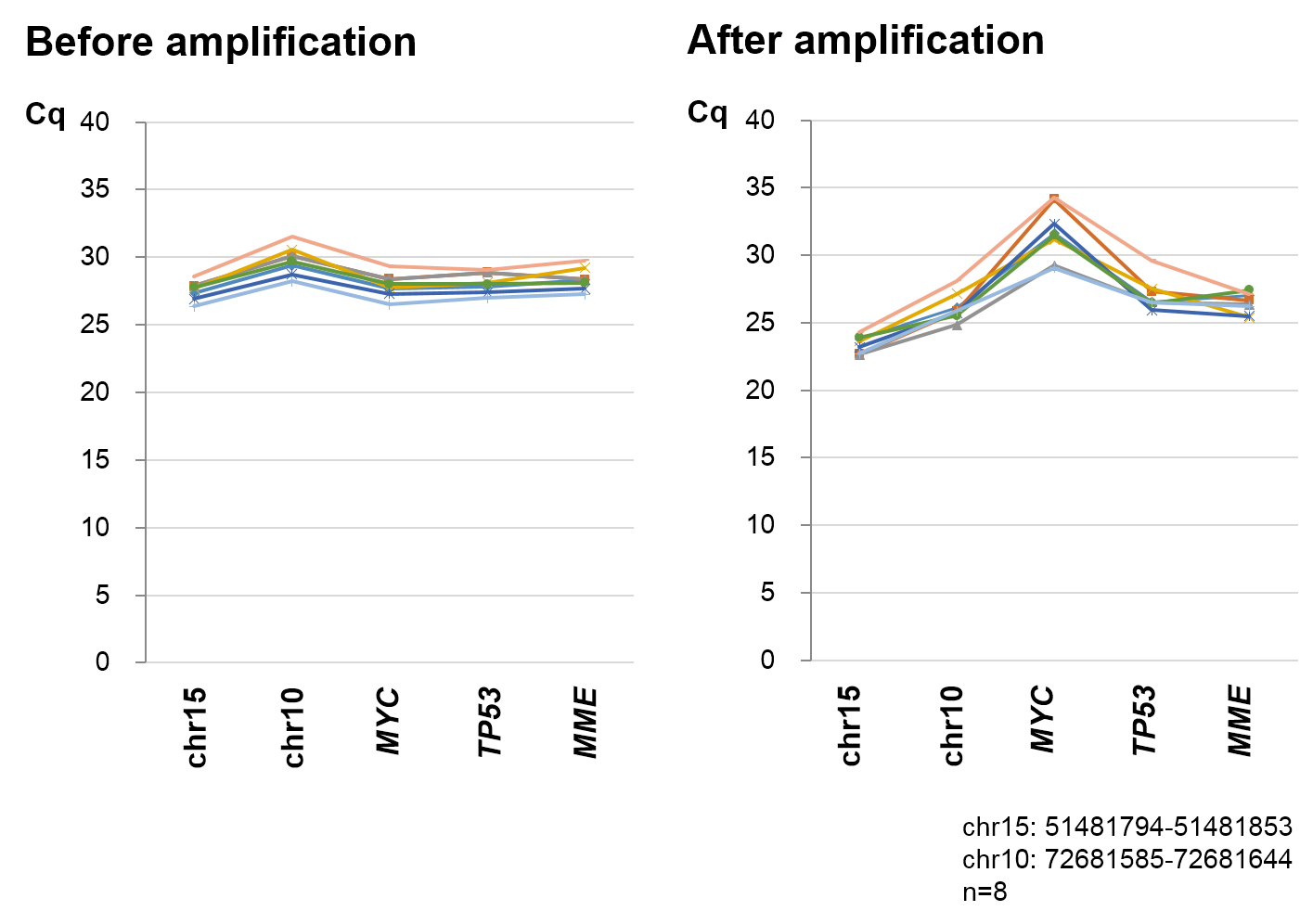

Supplement: Additional file 2: Figure S1. — Plot of PCR cycle number, at which the PCR products exceed the threshold (Cq). The Cq value profiles of 5 genes are shown for 8 reference samples before (a) and after (b) whole genome amplification. The genes used are markers of chromosome 15 and 10 (#51481794-51481853 and # 2681585–72681644, respectively), MYC, TP53, and MME. [file 12920_2015_80_MOESM2_ESM.png]

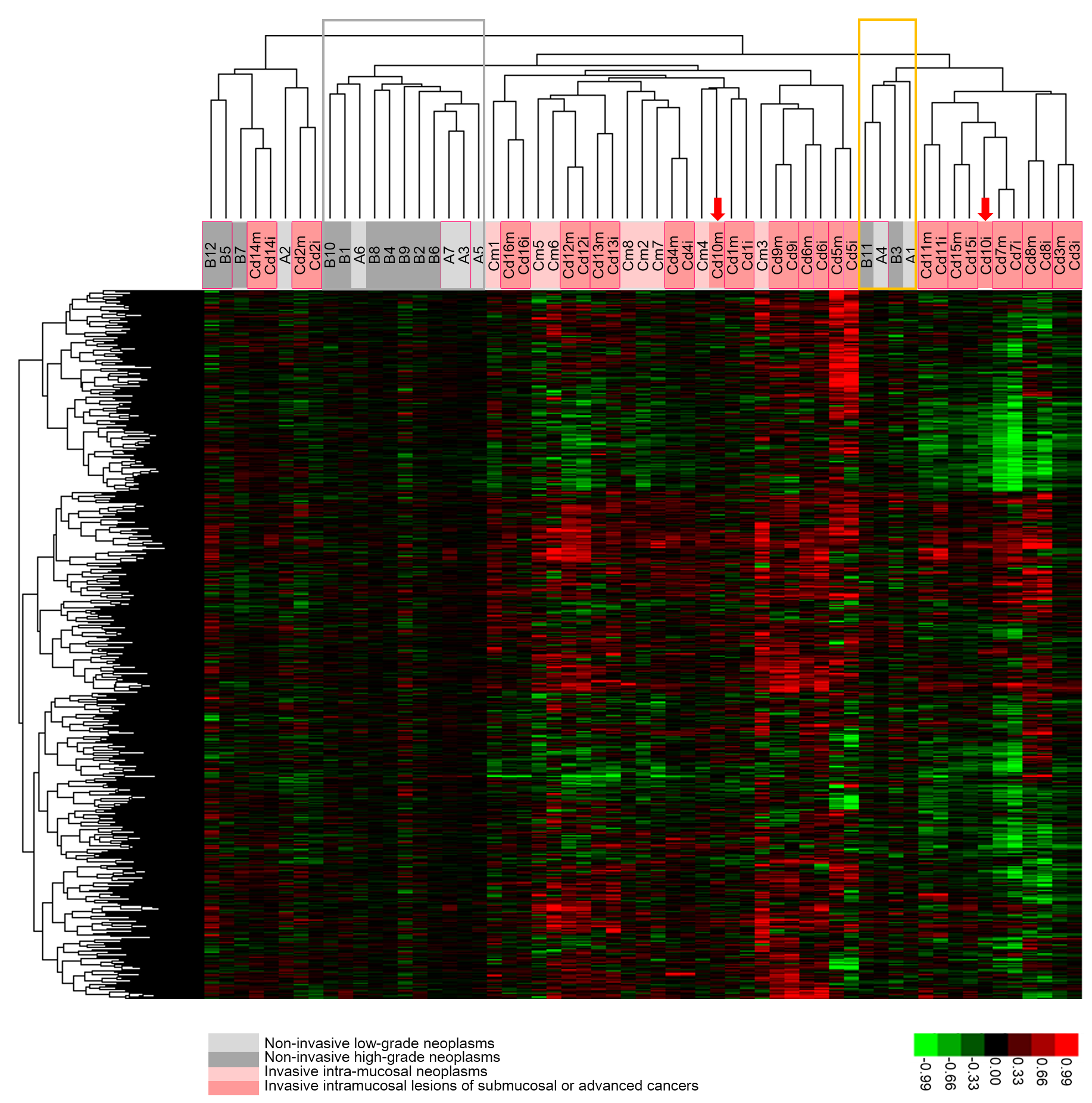

Supplement: Additional file 3: Figure S2. — Two-dimensional supervised cluster analysis using 43 mucosal and 16 invasive–part samples. To compare the copy-number profile of mucosal and invasive parts of individual tumours in the clustering analyses with varying size-dependent gene numbers, 16 samples from invasive parts of Cd tumours were added to the 43 mucosal samples. Following “Cd”, the sample numbers with and without “m” indicate intramucosal and extramucosal parts, respectively. (a) Clustering dendrograms with varying gene numbers, from 9,615 genes containing ≥2 probes to 373 genes containing ≥10 probes. Thick red frames are pairs of mucosal and invasive samples from identical tumours. These pairs are consistently neighbouring except for 1 pair of samples marked with closed red arrows. Of the pairs of concurrent tumours that were located separately from each other in the single patient and marked with a pair of numbers under the sample name, the pairs that are located in neighbouring positions in the dendrogram are marked with thin red frames; those in split positions are marked with pairs of closed or open black arrows. (b) A heat map of the clustering that corresponds to the upper right dendrogram of (a), using 2,863 genes of ≥4 probes. Grey and yellow squares indicate the samples of the stable and the intermediate clusters, respectively. The samples without a frame belong to the unstable cluster. [file 12920_2015_80_MOESM3_ESM.zip › 12920_2015_80_MOESM3b_ESM.png]
